# Interactions that define the arrangement of sugar-binding sites in BDCA-2 and dectin-2 dimers

**DOI:** 10.1093/glycob/cwae082

**Published:** 2024-10-03

**Authors:** Yu Liu, Jong-Won Kim, Hadar Feinberg, Nikeel Cull, William I Weis, Maureen E Taylor, Kurt Drickamer

**Affiliations:** Department of Life Sciences, Sir Ernst Chain Building, Imperial College, Exhibition Road, London SW7 2AZ, United Kingdom; Department of Life Sciences, Sir Ernst Chain Building, Imperial College, Exhibition Road, London SW7 2AZ, United Kingdom; Departments of Structural Biology and Molecular and Cellular Physiology, Fairchild Building, Stanford University School of Medicine, 299 Campus Drive West, Stanford, CA 94305, United States; Department of Life Sciences, Sir Ernst Chain Building, Imperial College, Exhibition Road, London SW7 2AZ, United Kingdom; Departments of Structural Biology and Molecular and Cellular Physiology, Fairchild Building, Stanford University School of Medicine, 299 Campus Drive West, Stanford, CA 94305, United States; Departments of Structural Biology and Molecular and Cellular Physiology, Fairchild Building, Stanford University School of Medicine, 299 Campus Drive West, Stanford, CA 94305, United States; Department of Life Sciences, Sir Ernst Chain Building, Imperial College, Exhibition Road, London SW7 2AZ, United Kingdom

**Keywords:** carbohydrate-binding protein, crystal structure, glycoprotein, lectin, receptor

## Abstract

The sugar-binding receptors dectin-2 and blood dendritic cell antigen 2 (BDCA-2) bind oligosaccharide ligands through extracellular carbohydrate-recognition domains (CRDs) and initiate intracellular signaling through Fc receptor γ adapters (FcRγ). Dectin-2 stimulates macrophages in response to pathogen binding while BDCA-2 modulates cytokine production in plasmacytoid dendritic cells. The oligomeric states of these receptors and the orientations of their CRDs have been investigated by analysis of a naturally occurring disulfide-bonded variant of BDCA-2 and by replacement of transmembrane domains with N-terminal dimerization domains to create extracellular domain dimers of both dectin-2 and BDCA-2. Analysis of these constructs, as well as previously described crystal structures of the CRDs from these proteins and a novel structure of an extended version of the extracellular domain of dectin-2, showed that there is only limited interaction of the CRDs in the dimers, but interactions can be stabilized by the presence of the neck region. The resulting orientation of sugar-binding sites in the dimers would favor crosslinking of multiple dimers by oligosaccharide ligands, causing clustering of FcRγ to initiate signaling.

## Introduction

Macrophage dectin-2 and blood dendritic cell antigen 2 (BDCA-2), together with the macrophage receptors mincle and MCL (macrophage C-type lectin), are signaling receptors that make up the dectin-2 family of structurally related C-type lectins. Each of these receptors consists of an N-terminal short cytoplasmic sequence and transmembrane domain linked to a C-terminal C-type carbohydrate-recognition domain (CRD) by a short neck region (Taylor and Drickamer 2015). Because these receptors lack intracellular signaling motifs, association with an Fc receptor γ adapter (FcRγ) adapter molecule is required for signaling initiation (Kerscher et al. 2013). The cytoplasmic domain of the adapter contains an immuno-tyrosine activation motif (Berry and Call 2017). When the receptors are activated by binding to glycan ligands, the associated FcRγ adapters initiate either a pro-inflammatory signaling pathway through Syk/CARD9/Bcl-10/MALT1 or an anti-inflammatory signaling pathway through Syk/BLNK/PLCγ2 (Cao et al. 2007; Drummond et al. 2011).

Dectin-2, also designated CLEC-6A, is found on the surface of dendritic cells, activated macrophages and inflammatory monocytes (Fernandes et al. 1999; Kanazawa et al. 2004; Gavino et al. 2005; Taylor et al. 2005) and binds the Manα1-2Man disaccharide, which is a common epitope in mannans on the surface of pathogenic yeast such as *Candida albicans* (Saijo et al. 2010; Feinberg et al. 2017). Dectin-2 activation increases production of pro-inflammatory cytokines in response to *C. albicans* infection and drives Th1/Th17 differentiation to combat fungal infection (Robinson et al. 2009; Saijo et al. 2010).

BDCA-2 is found only on the surface of plasmacytoid dendritic cells, which produce a high level of type I interferon in response to viral infection (Dzionek et al. 2000; Dzionek et al. 2001). BDCA-2 binds to glycans containing the Galβ1-3/4GlcNAcβ1-2Man epitope, which are commonly found on a subset of serum proteins, such as α_2_-macroglobulin and immunoglobulins A1, A2, G and M (Jégouzo et al. 2015; Kim et al. 2018). BDCA-2 initiates a pathway leading to inhibition of the type I interferon response and has the potential to suppress inflammation in autoimmune diseases, such as systemic lupus erythematosus (Dzionek et al. 2001; Cao et al. 2007; Furie et al. 2019).

**Fig. 1:**
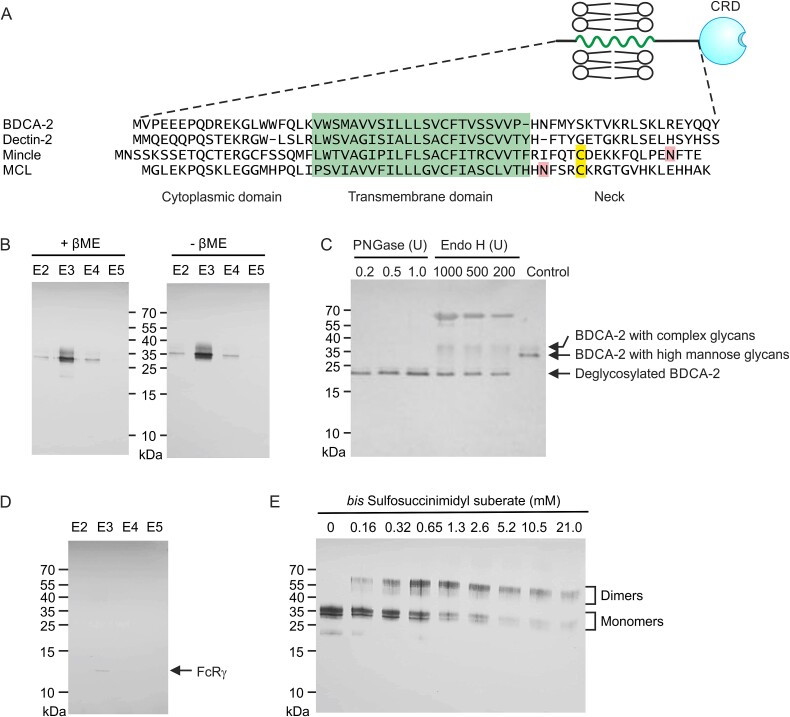
SDS-polyacrylamide gel electrophoresis of noncovalent BDCA-2 dimers. A Alignment of N-terminal sequences of BDCA-2, dectin-2, mincle and MCL. Transmembrane domains are highlighted in *green* and cysteine residues and potential asparagine-linked glycosylation sites in the neck region are highlighted in *yellow* and *pink*, respectively. B) Western blot of affinity-purified BDCA-2 from HEK cells expressing the full-length protein. Cell lysis solution, contained *N*-ethylmaleimide to prevent formation of artifactual disulfide bonds following extraction of BDCA-2 from the membrane. BDCA-2 was isolated on a 1-mL column of desialylated egg glycopeptide. Aliquots of 1-mL fractions eluted with EDTA were examined in the presence and absence of reducing agent 2-mercaptoethanol (βME). C) Western blot of BDCA-2 digested with endoglycosidases. Purified BDCA-2 was denatured and digested with endoglycosidase H (EndoH) and protein N-glycanase (PNGase). Recombinant endoglycosidase H reacts with the detection reagents and appears as a band near 70 kDa. D) Western blot showing copurification of FcRγ with BDCA-2. Fractions from egg-glycopeptide affinity column, as in (B), were separated in the presence of reducing agent. E) Western blot of cross-linked BDCA-2. Fractions containing BDCA-2 were pooled and reacted with various concentrations of bifunctional reagent for 30 min before quenching with 2x sample buffer containing reducing agent. In (B–E), proteins were detected with either anti-BDCA-2 or anti-FcRγ antibodies followed by alkaline phosphatase-conjugated protein A.

The intracellular and transmembrane portions of dectin-2 and BDCA-2 are closely related to each other, while mincle and MCL are more similar to each other. However, the neck regions, which lie between the membrane and the CRDs, are not highly conserved and do not have obvious structural motifs. For example, N-linked glycosylation sites and cysteine residues are present in the neck portions of mincle and human MCL, but not in human dectin-2 or BDCA-2 (Fig. 1A).

In the present work, characterization of the dimeric forms of these proteins stabilized by appended coiled-coil dimerization domains, along with analysis of crystal structures of the CRDs and locations of interchain disulfide bonds, provides a basis for modelling the organization of the dimers and how they might interact to initiate signaling.

## Results

### BDCA-2 forms a non-covalently linked dimer

Comparison of the sequences of the neck regions of BDCA-2, dectin-2 and mincle shows that the neck region of mincle contains a cysteine residue that is not present in dectin-2 or BDCA-2 (Fig. 1A). This cysteine residue in mincle forms an interchain disulfide bond to create covalently linked dimers (Liu et al. 2024). In order to determine the oligomeric state of BDCA-2 in cells, full-length BDCA-2 and FcRγ were co-expressed in human embryonic kidney (HEK) cells. Analysis of BDCA-2 purified from lysates of the transfected HEK cells by affinity chromatography on a column bearing Galβ1-3/4GlcNAcβ1-2Man-terminated glycopeptide showed that wild-type BDCA-2 does not form interchain disulfide bonds (Fig. 1B). The presence of two bands reflects partial processing of the glycans from high mannose to complex structures (Fig. 1C), which suggests that at least some of the BDCA-2 is transported out of the Golgi. Co-purification of the FcRγ adapter also indicates that the BDCA-2 forms a complex with the signaling adapter (Fig. 1D). Although there are no interchain disulfide bonds, chemical crosslinking indicates that BDCA-2 forms a dimer that is not disulfide-linked (Fig. 1E).

### BDCA-2 dimers crosslinked by a cysteine in the neck domain

**Fig. 2:**
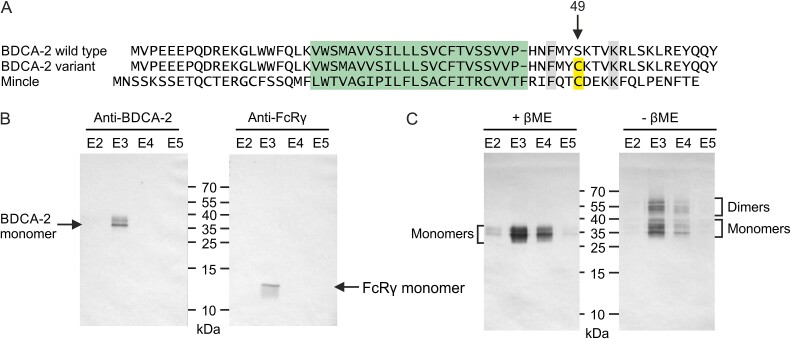
Analysis of BDCA-2 with a cysteine residue in the neck region. A) Alignment of N-terminal sequences of mincle, BDCA-2 and the BDCA-2 Ser49Cys variant. Transmembrane domains are highlighted in *green* and the amino acids on either side of position 49 are aligned as indicated by *grey* shading. Key cysteine residues are highlighted in *yellow*. B) SDS-polyacrylamide gel analysis of FcRγ co-purification with BDCA-2 Ser49Cys variant. Following affinity chromatography on immobilized desialylated egg glycopeptide, BDCA-2 and FcRγ from transfected cells were detected by western blotting. C) Disulfide-linked dimers of Ser49Cys form of BDCA-2 identified by SDS-polyacrylamide gel electrophoresis. BDCA-2 purified by affinity chromatography was separated on SDS-polyacrylamide gels and detected with anti-BDCA-2 antiserum.

Screening of the human single nucleotide polymorphism database led to identification of SNP rs143790567 in the BDCA-2 gene that leads to the change Ser49Cys in a single individual. A cysteine residue at this position would be comparable to the cysteine residue in the neck region of mincle that forms an interchain disulfide bond (Liu et al. 2024), suggesting that the Ser49Cys version of BDCA-2 might also form an interchain disulfide bond (Fig. 2A). A full-length construct of the BDCA-2 Ser49Cys variant was created for co-transfection with FcRγ into HEK cells. Affinity purification of the BDCA-2 Ser49Cys variant shows unperturbed FcRγ copurification (Fig. 2B). The two bands of BDCA-2 represent forms with high mannose and complex glycans. Comparison of reducing and non-reducing gels shows that the BDCA-2 Ser49Cys variant forms a disulfide-linked dimer that is dissociated in the presence of 2-mercaptoethanol (Fig. 2C). The ability to form such a disulfide bond provides evidence that, in the BDCA-2 dimer, the two copies of the neck region around residue 49 are close to each other.

### Dimeric ECD fragments of BDCA-2 produced in a bacterial expression system

**Fig. 3:**
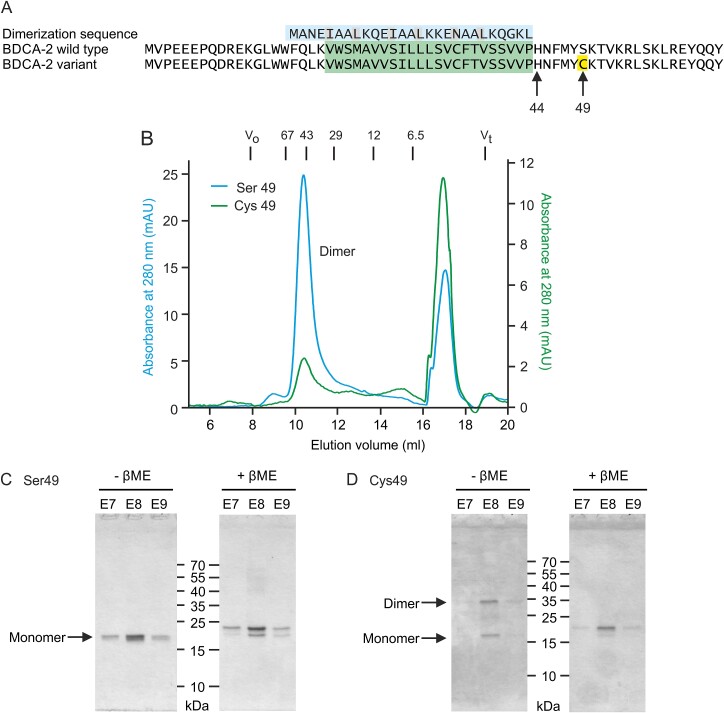
Characterization of human BDCA-2 extracellular domain constructs fused to 3-heptad dimerization domains. A) Extracellular domain fragments with dimerization sequence, shaded *blue*, replacing transmembrane domain, shaded *green*. Core residues in the heptad repeats of the dimerization domain are shaded *pink*. B) Superdex S75 gel filtration analysis of each affinity-purified construct. Elution positions of standards are indicated in kDa at the top. C and D) SDS-polyacrylamide gel electrophoresis of reference and variant forms of BDCA-2 extracellular domain eluted from a 5-mL desialylated egg glycopeptide column with EDTA. Fraction of 1 mL were collected. Gels were stained with Coomassie blue.

In order to replicate BDCA-2 dimerization in a soluble form, the transmembrane and intracellular domains of the BDCA-2 Ser49Cys variant and of wild type BDCA-2 were replaced with a 3-heptad coiled-coil dimerization sequence (Fig. 3A) (Harbury et al. 1993; Doñate et al. 2000; Fletcher et al. 2012; Woolfson et al. 2012). The fusion proteins were expressed in a bacterial system analogous to that previously used for expression of the CRD from BDCA-2 (Jégouzo et al. 2015). The purified proteins had apparent molecular weights of approximately 45 kDa by gel filtration, consistent with the expected molecular weight of dimeric BDCA-2 extracellular domain with appended dimerization domain (Fig. 3B). The presence of the neck cysteine residue in the Ser49Cys variant results in formation of disulfide-linked dimers that can be dissociated by 2-mercaptoethanol (Fig. 3C and D). In a membrane, association of the neck regions would probably be enhanced as a result of the orientation of the polypeptides which would place the neck cysteine residues at a common distance from the membrane. Thus, the fused dimerization domains are acting as a replacement of the transmembrane domain. These results provide further evidence that the two copies of the neck region around residue 49 are close to each other.

**Fig. 4:**
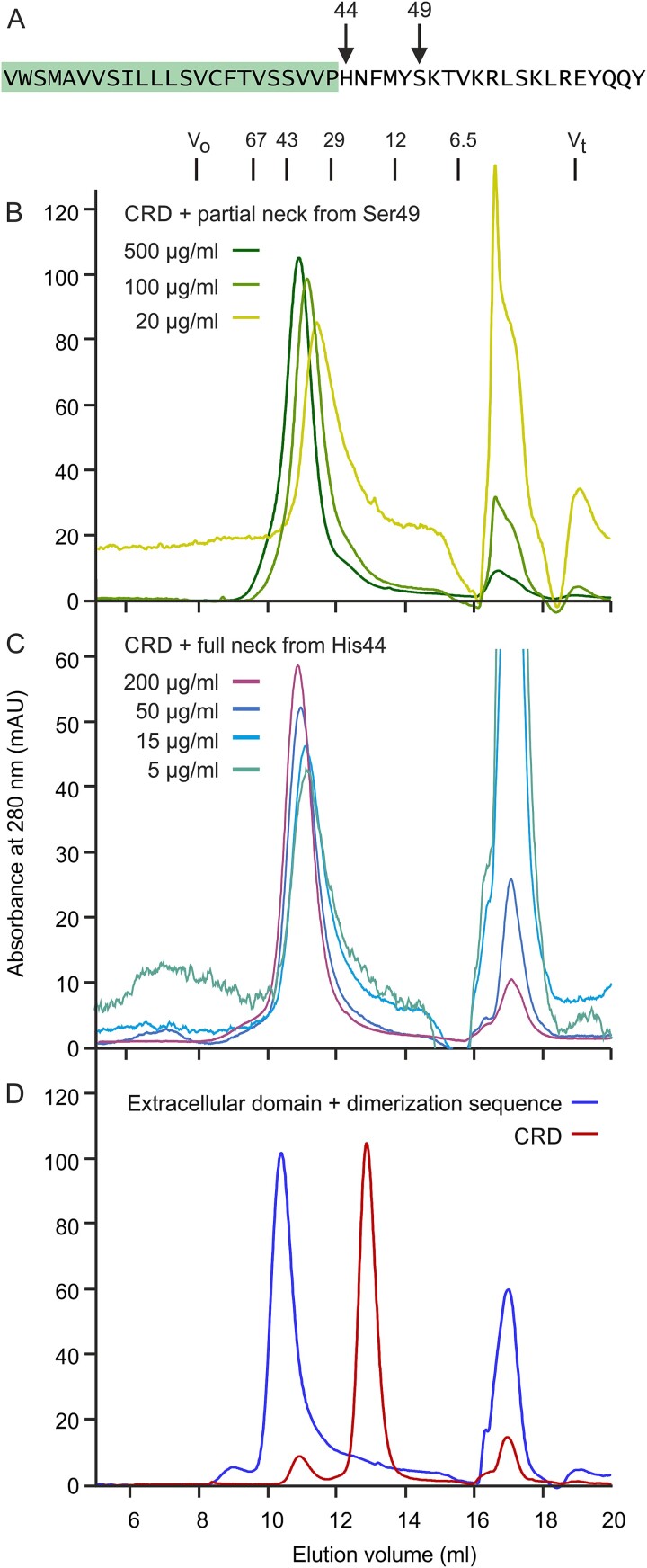
Superdex S75 gel filtration analysis of BDCA-2 extracellular domain without dimerization sequence. A) Sequence showing the starting positions of the expressed fragments, with the transmembrane domain shaded. B) CRD with partial neck domain at various concentrations. C) CRD with full neck domain at various concentrations. D) CRD alone and with neck domain and dimerization domain. In (B and C), absorbance values for diluted samples have been multiplied by the dilution factor for comparison.

Analysis of fragments of BDCA-2 beginning at residue His 44 and Ser49, with partial or full neck domains, provided further evidence for a role of this domain in stabilizing BDCA-2 dimers. On a gel filtration column, these fragments elute at apparent molecular weights approaching the expected molecular weight of dimers (Fig. 4A–C). Compared to the monomeric CRD and the dimer formed with the fused dimerization domain (Fig. 4D), the peaks are broader, and they shift to a lower molecular weight with dilution. These features indicate that the complex is unstable and is probably an equilibrium between monomeric and dimeric species. Inclusion of the full neck domain appears to result in greater stabilization of the dimer.

### Potential dimer interactions observed in crystal structures

**Fig. 5:**
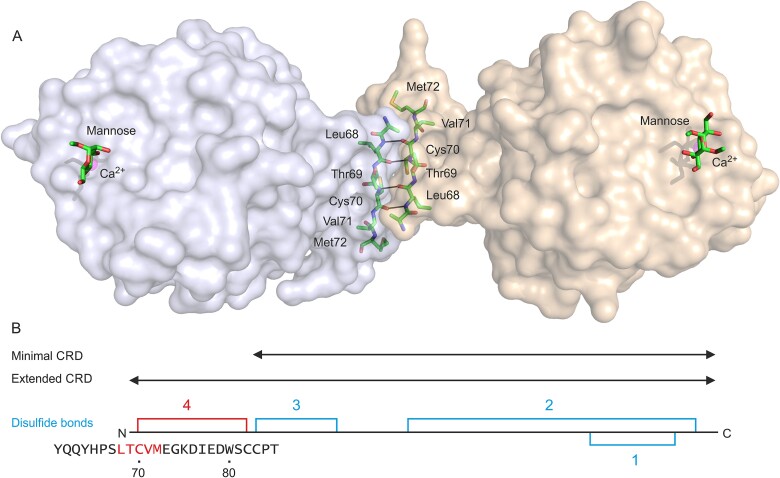
View of BDCA-2 dimer interface. A) A possible interface identified between chain A of the original unit cell (*left*) and chain B of a symmetry mate generated by symmetry operator x + 1/2, y + 1/2, z + 1/2, shifted −1 unit along the y-axis (*right*). Hydrogen bonds between the N-termini of the two subunits are shown. In this view, the two sugar-binding sites, occupied by a mannose residue, are facing out of the page. Image was generated using PDB entry 4ZES. B) Arrangement of disulfide bonds in C-type CRDs. Disulfide bonds 1 and 2 are found in all C-type CRDs and bond 3 found in many of the CRDs. The extra disulfide bond 4 is found in the dectin-2 family receptors. The dimer interface residues LTCVM in BDCA-2 are highlighted in *red*.

Although the CRD of BDCA-2 on its own behaves as a monomer in gel filtration, the fact that a short neck domain can stabilize dimers suggests that there are likely to be additional interactions between the CRDs. When existing crystal structures of the CRD of BDCA-2 were examined, potential interactions of the N-terminal ends of the CRDs that might reflect interactions in a dimer were observed between symmetry mates. The CRD crystals contain two copies of the CRD in the asymmetric unit, which are designated chain A and chain B (Jégouzo et al. 2015). A possible BDCA-2 dimer interface can be observed between chain A and chain B of a symmetry mate (Fig. 5A). Interface surface areas of 351 and 378 Å^2^ were calculated for the two subunits. These numbers are not identical, because the two copies of BDCA-2 that form the dimer being visualized are not related by a crystallographic symmetry axis. The area of this interface is small compared to the size of the CRDs, which is consistent with the observation that the neck is also required to stabilize the dimer.

Interestingly, the dectin-2 family receptors are amongst the few that have an N-terminal extension to the C-type CRD, which contains an extra intrachain disulfide bond (Fig. 5B) (Feinberg et al. 2016; Feinberg et al. 2017). Structural analysis shows that this extra disulfide bond locks a stretch of amino acids in a β-hairpin structure, which extends these CRDs and forms the region involved in the dimer interface (Fig. 5B). Taken together, the results suggest that BDCA-2 dimers result from direct interactions of the CRDs that are stabilized by portions of the neck domain brought into close proximity by the adjacent transmembrane sequences.

### Dimerization interface of dectin-2 observed in crystal structures

**Fig. 6:**
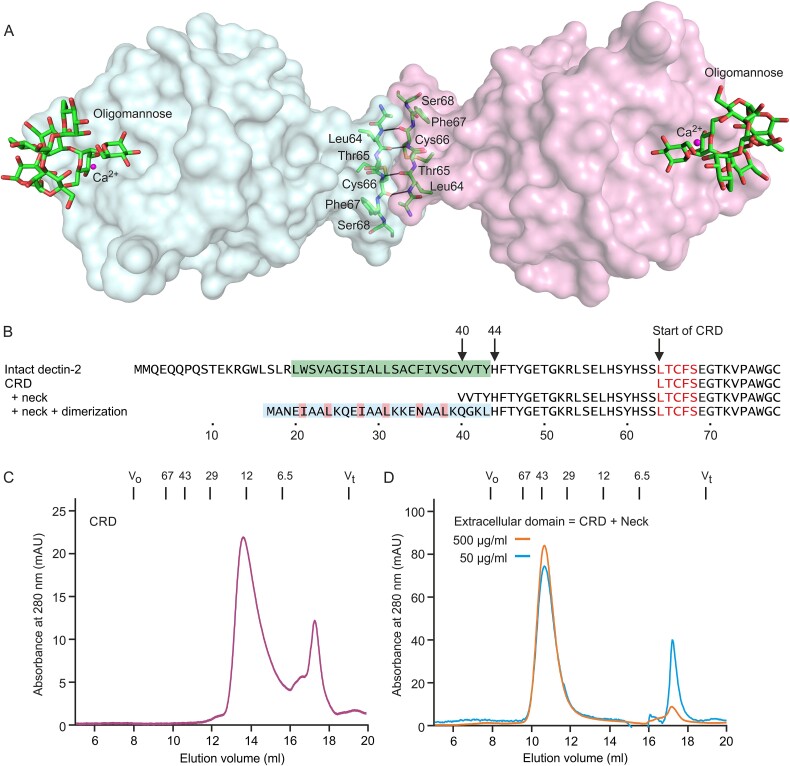
Dectin-2 dimer interface. A) Candidate interface between the original copy of the dectin-2 CRD (*left*) and a symmetry mate generated by symmetry operator −X, Y, −Z + 1/2, shifted +1 unit along the x axis (*right*). Bound oligosaccharide ligands are shown facing out of the page. Hydrogen bonds formed between the N-termini of the CRDs are shown. Image was generated using PDB entry 5VYB. B) Extracellular domain fragment produced in bacteria was initiated at residue Val40, while fusion with dimerization sequence was initiated at His44. Transmembrane domain is shaded *green* and dimerization domain is shaded *blue*, with core residues in the heptad repeats in *pink*. Residues that interact in the dimer of CRDs are shown in *red*. C) Superdex S75 gel filtration analysis of dectin-2 CRD. D) Superdex S75 gel filtration analysis of dectin-2 extracellular domain. The protein was analyzed following serial dilutions. Absorbance values for diluted samples have been multiplied by the dilution factor for comparison.

A potential dimer interface very similar to the one observed in BDCA-2 can be identified in the crystal structure of dectin-2 (Feinberg et al. 2017). The crystals contain a single dectin-2 CRD in the asymmetric unit, so the proposed dimer interface is formed with one of the symmetry mates (Fig. 6A). The surface area of the interface is 366 Å^2^. This dimer is strikingly similar to that seen for BDCA-2, in spite of the different crystal packing and the fact that the subunits of the dectin-2 dimer are related by a crystallographic symmetry axis while those in the BDCA-2 dimer are not.

Gel filtration analysis indicates that the CRD of dectin-2 exists as a monomer (Fig. 6C) while the full extracellular domain of dectin-2 forms a dimer (Fig. 6D). The elution position of the extracellular domain does not change as the protein is diluted, indicating that the dimer is stable. Thus, in contrast to BDCA-2, the presence of the full neck domain in the expressed extracellular domain of dectin-2 is sufficient to stabilize the dimeric state.

### Stabilization of dectin-2 dimers with dimerization domains

**Fig. 7:**
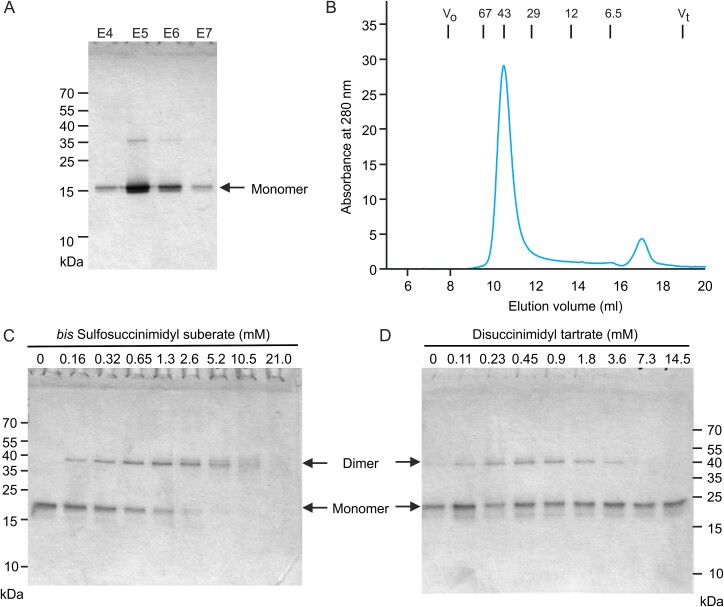
Characterization of dectin 2 extracellular domain linked to 3-heptad coiled-coil dimerization domain. A) SDS-polyacrylamide gel analysis of 0.5-mL fractions eluted with EDTA from a 2-mL mannose-Sepharose affinity column. Gel was run in the absence of 2-mercaptoethanol and stained with Coomassie blue. B) Superdex S75 gel filtration of purified extracellular domain. Sample was dialyzed against water, lyophilized and dissolved in gel-filtration buffer. C and D) Crosslinking of extracellular domain with bifunctional reagents bis-sulfosuccinimidyl suberate and disuccinimidyl tartrate. Reactions were stopped by adding 2X sample buffer containing 2% 2-mercaptoethanol and were separated on 17.5% SDS-polyacrylamide gels that were stained with Coomassie blue.

In spite of the stability of the extracellular domain dimer of dectin-2, it did not form crystals. In an attempt to stabilize the conformation of the neck domain, the dectin-2 extracellular fragment beginning with residue His44 was fused to the same 3-heptad dimerization domain used with BDCA-2 (Fig. 6B). This fusion product elutes in gel-filtration at the molecular weight of 44 kDa expected for a dimer (Fig. 7A and B). The formation of dimers was confirmed by chemical crosslinking with reagents that bridge 6.4 and 11.4 Å (Fig. 7C and D).

### CRD interfaces in crystals of dimerized dectin-2

**Fig. 8:**
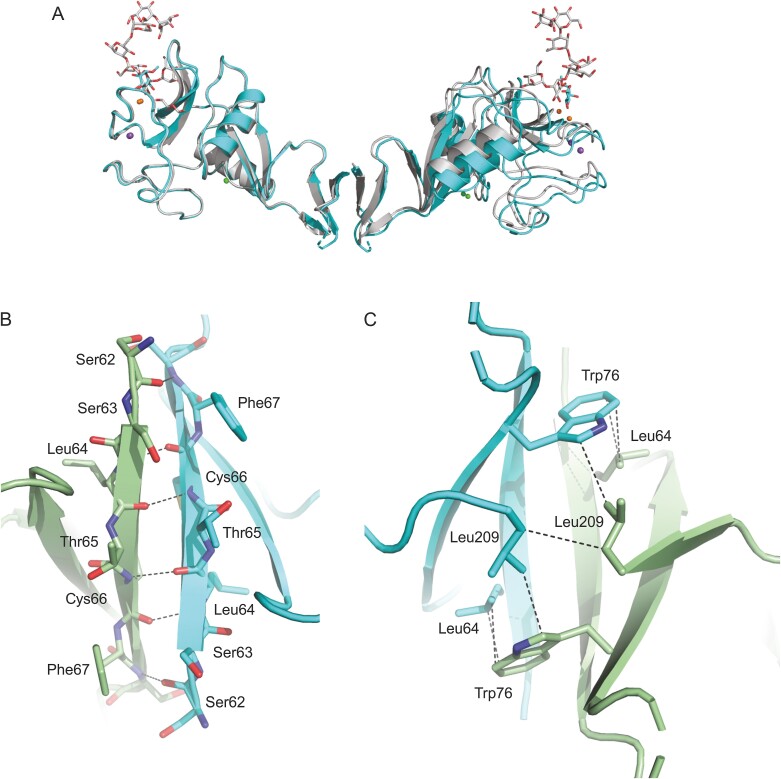
Structure of dectin-2 in crystals made with dectin-2 CRD plus neck and dimerization domain. A) Comparison of dectin-2 structures. The previously described structure of the dectin-2 CRD in complex with a mannose oligosaccharide (PDB entry 5VYB) is in *grey* and the new structure derived from crystals of the CRD with neck and dimerization domain complexed with α-methyl mannoside is in *cyan*. Ca^2+^ at the sugar-binding sites are shown in *orange*, supplemental Ca^2+^ are shown in *green* and bound Na^+^ is shown in *purple*. B) Hydrogen bonds between the extended *N*-terminal sequences. C) Additional contacts across the dimer interface.

Analysis of new crystals formed by the dectin-2 dimer fused to the dimerization domain provided further insight into the interface structure (Fig. 8). The geometry of the CRDs in crystals, which contain two dectin-2 polypeptides in the asymmetric unit, is almost identical to that observed for the isolated CRD. Two additional amino acids, SerSer at the N-terminus of each subunit, are visible in the new crystal structure. The extended interface in the new crystal expands the 4-stranded antiparallel β sheet across the dimer interface observed for the BDCA-2 and dectin-2 CRDs, contributing to a more extensive surface area of 453 Å^2^.

The arrangement of the dimerized dectin-2 extracellular domain shows that the geometry of the CRDs in the dimer matches closely the arrangement seen for the isolated CRD, suggesting that the dimerization domain stabilizes the interface observed between isolated CRDs, but does not significantly alter its geometry. The finding of a similar interface between the CRDs in a completely different crystal form is also consistent with the suggestion that this represents an interface in the native dimers. Overlay of the extracellular domain dimer and the CRD dimer does show a very small amount of flexibility in the relative positions of the CRDs (Fig. 8A). The extended N-terminus introduces two more hydrogen bonds in the β-strands connecting the monomers, between Ser62 and Ser68 (Fig. 8B). Additional reciprocal packing interactions can be found between Trp76 of one monomer to Leu209 and Leu64 of the other and between the Leu209 residues (Fig. 8C).

### Conformation of the neck domain

**Fig. 9:**
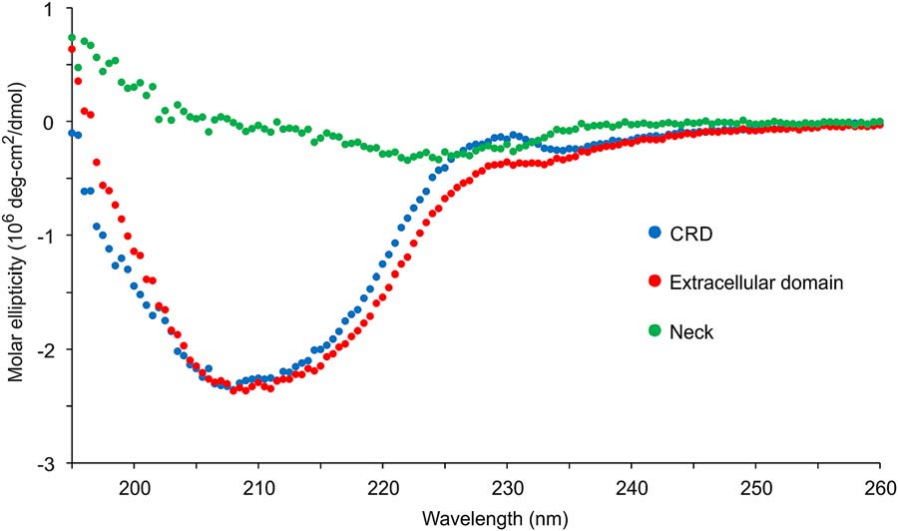
Circular dichroism analysis of dectin-2 neck domain. The neck spectrum was obtained by subtracting the spectrum of the CRD from the spectrum of the CRD plus neck fragment starting at residue Val40 (Fig. 6B). Similar results were obtained in the presence of 2.5 mM CaCl_2_.

Since the dimerization domain is likely to form a coiled-coil, the fact it is absent from the crystal indicates that its position is not ordered in the crystal, which in turn suggests that the neck region of dectin-2 may be flexible. The conformation was investigated by comparing the circular dichroism spectra of the CRD and the extracellular domain (Fig. 9). The spectrum of the CRD is similar to spectra of other C-type CRDs (Jégouzo et al. 2013), but there is very little difference between the CRD and the extracellular domain. The difference spectrum, which would correspond to the neck domain, is almost flat and shows no evidence of regular secondary structure, ruling out the possibility of a coiled-coil of α-helices in the neck, in contrast to what has been observed for other membrane-bound C-type lectins (Jégouzo et al. 2013).

## Discussion

**Fig. 10:**
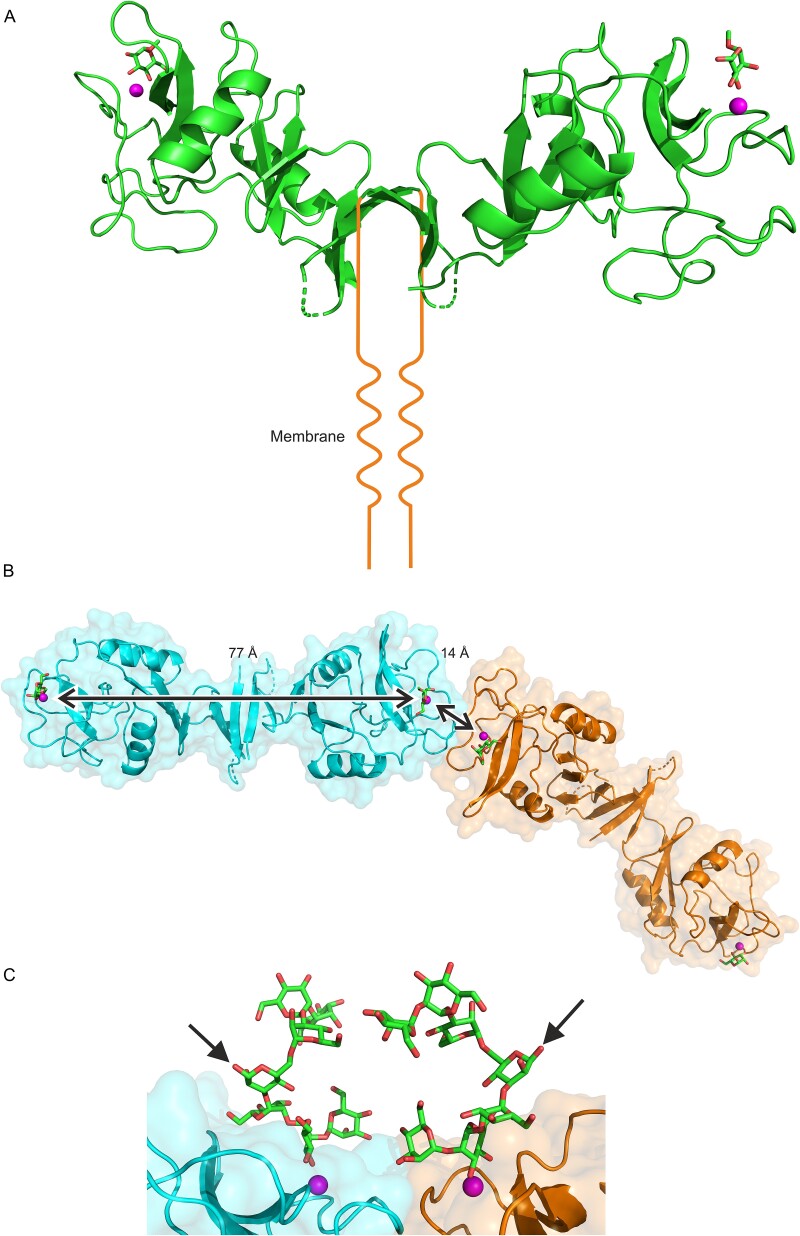
Summary of possible organization of dectin-2 dimers on the cell membrane. A) Side view of a proposed dectin-2 dimer showing its potential orientation relative to the membrane. Stretches of amino acids that loop around to the membrane are shown as orange lines. Ca^2+^ at the sugar-binding sites are shown in magenta. B) An example of a possible interaction of two dimers, shown in top view looking down on the membrane. The arrangement is hypothetical, but the minimal electrostatic potential on the surfaces brought together in the dimer-dimer interface makes the close contact shown plausible. Spacings of the Ca^2+^ at the sugar-binding sites, within a dimer and between adjacent dimers, are indicated. Bound α-methyl mannosides in the ligand-binding sites are shown facing out of the page. C) Superposition of two high mannose oligosaccharides bound to the CRDs from two dectin-2 dimers in the orientation shown in (B). The arrangement of the bound oligosaccharides are taken from PDB entry 5VYB and the structure is viewed from the side. Arrows indicate the reducing ends of the oligosaccharides.

Interactions between the CRDs observed in crystal structures of the CRDs from both dectin-2 and BDCA-2 provide a basis for modelling of the structures of the receptor dimers (Fig. 10A). Although the proposed model for dimer arrangement remains speculative, it is consistent with multiple lines of evidence. Importantly, the observed interactions between the CRDs are nearly identical in three different crystal forms, in two of which the dimer forms the asymmetric unit (Fig. 5A and Fig. 8) as well as one in which the dimer forms part of the crystal symmetry (Fig. 6A). The fact that the dimer interface involves hydrogen bonds between backbone atoms in a β sheet is consistent with the absence of extensive sequence conservation in the neck region (Fig. 1A).

Based on correlation of domain interface area with domain size (Jones and Thornton 1996), the relatively small interfaces in dectin-2 and BDCA-2 are consistent with the finding that the CRD-CRD interactions alone would not be sufficient to stabilize dimers. For BDCA-2, the importance of the neck domain in dimer formation is indicated by the demonstration that part of the neck is sufficient to form dissociating dimers and the fact that an interchain disulfide bond can be formed between the necks. Attachment of a coiled-coil dimerization domain may act as a substitute for the transmembrane domain by providing critical interactions beyond the neck region and aligning the CRDs from two subunits to form stable dimers. The results suggest that preformed dimers are a common feature of receptors in the dectin-2 family, including mincle in which dimers are stabilized by a disulfide bond (Liu et al. 2024).

The CRD-CRD dimer interfaces determine the orientation of the CRDs relative to each other. The two additional serine residues visible in the new dectin-2 dimer crystals described here project to the side, indicating that the neck could bend around the CRD-CRD interface to link to the transmembrane domain (Fig. 10A). These portions of the neck must come close to each other, to allow disulfide bond formation in the BDCA-2 variant, but the absence of density corresponding to either the remainder of the neck or the dimerization domain suggests the necks are likely to be flexible.

The proposed geometry of the BDCA-2 and dectin-2 dimers puts major constraints on how multivalent ligands would interact with these receptors. Binding of single oligosaccharides to two subunits in a dimer would be precluded, because the distance across the dimer is approximately 77 Å and would require a precise geometric fit to the two binding sites (Fig. 10B). The similar geometry in BDCA-2 and dectin-2 suggests that the relative orientation of the CRDs is not likely to be significantly changed by the ligands in order to accomodate binding to the two sites. The geometry of the dimer thus selects against binding of multivalent ligands to two subunits in a single dimer.

It is however possible to bring the binding sites in subunits of two different dimers within 14 Å of each other (Fig. 10B) and similar interactions could form in multiple different orientations. The crystal structure of dectin-2 with a bound high mannose oligosaccharide shows how individual CRDs interact with oligosaccharide ligands (Feinberg et al. 2017). Superposition of these bound oligosaccharides onto CRDs from two dimers arranged on a membrane surface as in Fig. 10B suggests that even when the binding sites come as close as 14 Å, they would likely be occupied by separate glycans attached to a protein or cell surface in order to initiate signaling by clustering of receptor dimers (Fig. 10C).

Synthetic ligands that cause clustering on cells could have utility in modulation of the immune response. For example, BDCA-2 stimulation is being targeted for suppression of interferon α/β secretion in systemic lupus erythematosus and other auto-immune diseases (Furie et al. 2019). Knowledge of receptor dimer geometry may facilitate design of bivalent or multivalent stimulatory ligands with appropriate spacing between binding epitopes. Such ligands would need to be tested on cells, since interaction of the bridging ligand would only need to stabilize dimer-dimer interactions in the constrained, two-dimensional environment of the membrane. In addition, the dectin-2 results suggest that multivalent binding with inappropriate geometry might be observed when the CRD is in solution and not on the cell surface.

## Materials and methods

### Analysis of BDCA-2 in transfected HEK cells

DNA coding for human BDCA-2 was cloned into expression vector pSF-CMV-EMCV-Zeo (Oxford Genetics) and DNA coding for human FcRγ was cloned into vector pIRESHyg2 (Clontech). Expression plasmids were purified with the NucleoBond Xtra Midi plasmid kit (Macherey-Nagel) following the manufacturer’s protocol and sterilized by ethanol precipitation. HEK 293 cells at ~90% confluence on 35 mm plates were transfected with 1.25 μg of each plasmid mixed with 3.75 μL of lipofectamine 3,000 reagent (Invitrogen) in 125 μL of OptiMEM (Invitrogen). After 48 h, cells were moved to Dulbecco’s Modified Eagle’s Medium containing 10% fetal calf serum, 10 U/mL penicillin–streptomycin, 300 μg/mL zeocin and 200 μg/mL hygromycin (Invitrogen). Single colonies of zeocin- and hygromycin-resistant cells were picked and transferred to a 24-well plate before being passaged into 25 or 75 cm^2^ flasks. The same procedures were used to generate HEK cells co-expressing FcRγ and human BDCA-2 with the mutation Ser49Cys.

Two 75-cm^2^ flasks of HEK BDCA-2 / FcRγ transfectants were rinsed twice with 10 mL of phosphate-buffered saline, harvested and lysed by homogenization in 5 mL of cold lysis buffer containing 0.15 M NaCl, 25 mM Tris-Cl pH 7.8, 2.5 mM CaCl_2_, 10 mM *N*-ethylmaleimide, 1% Triton X-100 (Roche), and 50 μL of protease inhibitor (Merck Millipore). Lysate was centrifuged at 16,000 g, 4 °C for 10 min and the supernatant was loaded onto a 1-mL desialylated egg-glycopeptide column (Jégouzo et al. 2015), which was rinsed with 5 mL of wash buffer (0.15 M NaCl, 25 mM Tris-Cl pH 7.8, 2.5 mM CaCl_2_, and 0.1% Triton X-100) and eluted in five 0.5-mL fractions with elution buffer (0.15 M NaCl, 25 mM Tris-Cl pH 7.8, 2.5 mM EDTA and 0.1% Triton X-100). Eluted fractions were precipitated by addition of trichloroacetic acid to 10% and incubated for 10 min at 4 °C. Precipitated fractions were centrifuged at 16,000 g for 5 min at room temperature, washed twice with 0.5 mL of ethanol:ether (1:1) and lyophilized. Pellets were dissolved in gel sample buffer with or without 1% 2-mercaptoethanol for SDS-polyacrylamide gel electrophoresis.

For endoglycosidase digestions, purified BDCA-2 was precipitated with trichloroacetic acid, resuspended in 0.5% SDS containing 40 mM dithiothreitol, and heated at 100 °C for 10 min. For PNGase treatment, the sample was incubated with 0.2, 0.5 or 1 unit of PNGase F (Roche) in 50 mM sodium phosphate pH 7.5 and 1% NP-40 at 37 °C for 15 min. For endoglycosidase H treatment, the sample was incubated with endoglycosidase H_f_ (New England Biolabs) in 50 mM sodium acetate, pH 6, at 37 °C for 15 min. Reactions were stopped by addition of equal volumes of 2X gel sample buffer containing 2% 2-mercaptoethanol and heating at 100 °C for 5 min.

### Expression and purification of extracellular domain fragments

Synthetic oligonucleotides (ThermoFisher) coding for a 3-heptad version of a dimerization domain based on the sequence designated CC-pIL-I17N (Fletcher et al. 2012) were appended to DNA coding for the extracellular regions (from His44) of human BDCA-2 and human dectin-2. Fusion proteins as well as the extracellular region of BDCA-2 starting at His44 or Ser49, the extracellular region of dectin-2 starting at Val40 and the CRDs of BDCA-2 and dectin-2 were expressed from pT5T plasmids in *Escherichia coli* strain BL21(DE3) and inclusion bodies were isolated as previously described (Jégouzo et al. 2015; Feinberg et al. 2017). After guanidine solubilization, BDCA-2 was renatured by dripping slowly into 4 volumes of dialysis buffer containing 0.5 M NaCl, 25 mM Tris-Cl 7.8, and 25 mM CaCl_2_ with stirring, followed by dialysis against three changes of the same buffer. Dectin-2 was renatured by slowly adding 4 volumes of dialysis buffer to the guanidine-solubilized material with stirring, followed by dialysis. After centrifugation and filtering through glass wool, dectin-2 was purified on mannose-Sepharose (Feinberg et al. 2017) and BDCA-2 was purified on a desialylated egg-glycopeptide agarose column (Jégouzo et al. 2015).

### Structural analysis

Fractions of affinity-purified dectin-2 extracellular domain with appended synthetic dimerization domain were pooled, dialyzed against water, lyophilized, and re-dissolved in water. The pH of the protein solution was adjusted to between 7 and 8 using 1 M NaOH and the protein concentration was determined by absorbance at 280 nm. Neutralized protein solution was re-lyophilized. The protein was dissolved at 6.3 mg/mL dectin-2 in a solution of 5 mM CaCl_2_, 10 mM Tris-Cl, pH 8.0, 25 mM NaCl, and 50 mM α-methyl mannoside and crystals were grown by sitting drop vapor diffusion at 22 °C using a Gryphon crystallization robot with a mixture of 0.15:0.15 μL of protein:reservoir solution in the drop. The reservoir solution contained 16% polyethylene glycol 3.35 K, 90 mM MES, pH 6.0 and 200 mM CaCl_2_. Freezing solution, containing 30% polyethylene glycol 3.35 K, 25 mM NaCl, 100 mM MES, pH 6.0, 200 mM CaCl_2_ and 50 mM α-methyl mannoside, was added to the drop before the crystal was frozen in liquid nitrogen for data collection.

Diffraction data were measured at 100 K on Beamline 12-1 at the Stanford Synchrotron Radiation Laboratory. The data were integrated with mosflm and scaled with AIMLESS (Battye et al. 2011; Evans and Murshudov 2013) to a maximum resolution of 2.36 Å (Table 1). The structure was solved by molecular replacement with the program Phaser (McCoy et al. 2007). The model used for molecular replacement was Protein Data Bank entry 5VYB, using chain A with two Ca^2+^, one Na^+^ and no water molecules or sugar. The molecular replacement solution confirmed that the space group was I2, with two monomers in the asymmetric unit. Model building and refinement were performed with Coot and PHENIX (Adams et al. 2002; Emsley and Cowtan 2004). Refinement included individual positional and isotropic temperature factor refinement and TLS refinement (Table 2).

**Table 1:** Crystallographic data statistics.

**Data**	**Dectin-2**
Symmetry	I2
Wavelength (Å)	0.97946
Unit cell lengths (Å)	a = 74.21 b = 65.03 c = 81.58 ß = 100.61
Resolution Å (last shell)	59.71–2.36 (2.45)
R_sym_ (last shell)^a^	11.4 (57.6)
Mn(I) half-set correlation CC(1/2)	0.993 (0.937)
Mean((I)/s(I)) (last shell)	6.9 (2.0)
% completeness (last shell)	96.0 (96.7)
Number of unique reflections	15,183
Average multiplicity (last shell)	5.3 (5.4)

^a^R_sym_ = 100x∑_h_∑_I_ (| I_i_(h) - <I(h)> |) / ∑_h_∑_i_ I_i_(h) where I_i_(h) = observed intensity, and <I(h)> = mean intensity obtained from multiple measurements.

**Table 2:** Crystallographic refinement statistics.

**Data**	**Refinement**
Number of reflections used for refinement	14,328
Reflections marked for R_free_	754
R_free_^a^	28.4
R_cryst_^a^	22.2
Average B factor (Å^2^)	54.7
Bond length rmsd (Å)	0.007
Angle rmsd (°)	0.887
Ramachandran plot: (% in each region)^b^ Preferred/ Allowed/ Outliers	95/5/0

^a^R and R_free_ = 100x∑_h_ |F_o_(h)-F_c_(h)| / ∑_h_F_o_(h), where F_o_(h) = observed structure factor amplitude and F_c_(h) = calculated structure factor amplitude for the working and test sets, respectively.

^b^As defined in Coot (Emsley and Cowtan 2004).

### Chemical crosslinking

For crosslinking experiments, BDCA-2 was purified from cells as described above, but using buffers containing 25 mM HEPES, pH 7.8, in place of Tris and dectin-2 extracellular domain with appended synthetic dimerization domain was re-purified by affinity chromatography on mannose-Sepharose using buffers containing 25 mM HEPES, pH 7.8 in place of Tris. Crosslinking reagents *bis*(sulfosuccinimidyl) suberate and disuccinimidyl tartrate (Pierce) were dissolved in water to 12 mg/mL and 50 mg/mL respectively, serially diluted with water and incubated with dectin-2 at room temperature for 30 min. Reactions were stopped by adding 2X sample buffer.

### Computational analysis

Molecular structures were visualized, analyzed and rendered for figures in PyMol. Buried surface area was analyzed using the PDBePISA software on the EMBL-EBI website (www.ebi.ac.uk/msd-srv/prot_int/pistart.html).

### Analytical procedures

SDS-polyacrylamide gel electrophoresis was performed on gels containing 17.5% acrylamide, followed by Coomassie blue staining or western blotting onto nitrocellulose. Rabbit polyclonal anti-BDCA-2 antibody was used at 1:5000 dilution as a primary antibody and protein A conjugated to alkaline phosphatase (Millipore) was used at 1:5000 dilution as a secondary reagent. For detecting FcRγ, rabbit polyclonal anti-FcRγ antibody (Millipore) was used at 1:2000 dilution as a primary antibody and protein A conjugated to alkaline phosphatase was used at 1:5000 dilution as a secondary reagent.

Gel filtration was performed on a 1 × 30 cm Superdex-S75 column (GE Healthcare Life Sciences) eluted with 10 mM Tris-Cl, pH 7.8, 100 mM NaCl and 2.5 mM EDTA at a flow rate of 0.5 mL/min at room temperature.

For circular dichroism analysis, samples were dialyzed against 25 mM NaCl, 5 mM Tris-Cl, pH 7.8 and analyzed at 0.1–0.2 mg/mL final concentration. Spectra were measured in a 1-mm quartz cuvette in an Applied Photophysics Chirascan spectropolarimeter at a band width of 1 nm, step size of 0.5 nm and 1 s per step. The spectra presented represent an average of ten scans.

## Data Availability

The atomic coordinates and structure factors (code 8ROV) have been deposited in the Protein Data Bank (http://wwpdb.org/).
